# Osteoconductive Properties of a Volume-Stable Collagen Matrix in Rat Calvaria Defects: A Pilot Study

**DOI:** 10.3390/biomedicines9070732

**Published:** 2021-06-25

**Authors:** Karol Alí Apaza Alccayhuaman, Stefan Tangl, Stéphane Blouin, Markus A. Hartmann, Patrick Heimel, Ulrike Kuchler, Jung-Seok Lee, Reinhard Gruber

**Affiliations:** 1 Department of Oral Biology, Dental School, Medical University of Vienna, Sensengasse 2a, 1090 Vienna, Austria; caroline7_k@hotmail.com (K.A.A.A.); cooldds@yuhs.ac (J.-S.L.); 2 Karl Donath Laboratory for Hard Tissue and Biomaterial Research, Division of Oral Surgery, School of Dentistry, Medical University of Vienna, 1090 Vienna, Austria; stefan.tangl@meduniwien.ac.at (S.T.); patrick.heimel@trauma.lbg.ac.at (P.H.); 3 Austrian Cluster for Tissue Regeneration, Medical University of Vienna, 1200 Vienna, Austria; 4 1st Medical Department, Ludwig Boltzmann Institute of Osteology at the Hanusch Hospital of OEGK and AUVA Trauma Centre Meidling, Hanusch Hospital, 1140 Vienna, Austria; stephane.blouin@osteologie.lbg.ac.at (S.B.); Markus.Hartmann@osteologie.lbg.ac.at (M.A.H.); 5 Ludwig Boltzmann Institute for Experimental and Clinical Traumatology, 1200 Vienna, Austria; 6 Department of Oral Surgery, Medical University of Vienna, 1090 Vienna, Austria; ulrike.kuchler@meduniwien.ac.at; 7 Department of Periodontology, Research Institute for Periodontal Regeneration, College of Dentistry, Yonsei University, Seoul 03722, Korea; 8 Department of Periodontology, School of Dental Medicine, University of Bern, 3010 Bern, Switzerland

**Keywords:** bone regeneration, volume-stable collagen matrix, collagen matrix, histology, qBEI

## Abstract

Volume-stable collagen matrices (VSCM) are conductive for the connective tissue upon soft tissue augmentation. Considering that collagen has osteoconductive properties, we have investigated the possibility that the VSCM also consolidates with the newly formed bone. To this end, we covered nine rat calvaria circular defects with a VSCM. After four weeks, histology, histomorphometry, quantitative backscattered electron imaging, and microcomputed tomography were performed. We report that the overall pattern of mineralization inside the VSCM was heterogeneous. Histology revealed, apart from the characteristic woven bone formation, areas of round-shaped hypertrophic chondrocyte-like cells surrounded by a mineralized extracellular matrix. Quantitative backscattered electron imaging confirmed the heterogenous mineralization occurring within the VSCM. Histomorphometry found new bone to be 0.7 mm^2^ (0.01 min; 2.4 max), similar to the chondrogenic mineralized extracellular matrix with 0.7 mm^2^ (0.0 min; 4.2 max). Microcomputed tomography showed the overall mineralized tissue in the defect to be 1.6 mm^3^ (min 0.0; max 13.3). These findings suggest that in a rat cranial defect, VSCM has a limited and heterogeneous capacity to support intramembranous bone formation but may allow the formation of bone via the endochondral route.

## 1. Introduction

Regenerative dentistry is an umbrella term for all procedures aiming to regain the form and function of oral tissues that are lost, mainly due to periodontal and periapical inflammatory osteolysis and the atrophy of the alveolar bone that occurs upon tooth extraction [1]. Today, dental implants can replace lost teeth with the remaining challenge to regenerate the lost bone, a process termed bone augmentation. Autologous bone, allografts, or bone substitutes and combinations thereof guide the newly formed bone to mechanically support the dental implants [2]. However, it also requires sufficient soft tissue to seal the augmented site and the dental implant towards the oral cavity [3,4]. The soft tissue dimension including its thickness can be a limitation to achieve a stable and esthetic outcome [5,6]. To overcome this limitation, a palate-derived connective tissue graft is used to regenerate the lost gingiva tissue, a process termed soft tissue augmentation [5,6]. To overcome the harvesting of the autografts, soft tissue substitutes were introduced to the field [7,8]. 

Collagen matrices have gained significant attention in the field of regenerative dentistry as soft tissue substitutes for connective tissue grafts [9]. This is based on the cumulative evidence that collagen matrices increase the soft tissue thickness and improve the contour around implants and teeth before prosthetic reconstructions [10,11,12]. Collagen matrices combine the clinical properties of connective tissue grafts, i.e., providing volume stability based on compression-resistant properties, with the ability to allow consolidation with the soft tissue at the augmentation site. Thus, a volume-stable collagen matrix (VSCM) enables the ingrowth of the adjacent soft tissues into the sponge-like porous structure [13,14]. Furthermore, collagen is the main extracellular matrix to support hard tissue regeneration. Therefore, it seems possible that the beneficial properties of VSCM are not limited to soft tissue augmentation but may also encompass osteoconductive properties.

Recent studies showed the capability of VSCM to consolidate with soft tissue in submucosal pouches of the canine maxilla. After around one month, a fibrous extracellular matrix filled the pores, and with time, the collagen matrix became fully integrated into soft tissue [13,14]. These studies support the claim that VSCM is conductive for soft tissue, comparable to what we consider as osteoconductive when grafts provide a surface for the newly formed bone during augmentation. Usually, there is a clear distinction between the biomaterials and grafts used for soft and hard tissue augmentation. However, this paradigm requires to be questioned, in particular, if it cannot be ruled out that a VSCM holds osteoconductive properties. 

First indications of possible osteoconductive behavior of VSCM stem from canine models showing the partial integration of VSCM into new bone [15,16]. Moreover, VSCM is cross-linked to increase the stiffness and stiffness is a parameter that was identified to affect chondrogenesis of gelatin scaffolds [17,18]. With respect to the defect site, calvaria has mesenchymal progenitors with an osteochondrogenic potential. Considering that collagen fibrils support the crystallization of hydroxyapatite during bone formation [19] it might be hypothesized that collagen-derived biomaterials in general, possess osteoconductive properties. Support for this hypothesis comes from observations that native collagen-rich membranes from the porcine peritoneum allow the accumulation of new bone [20,21]. These data support previous findings that new bone grows into the collagen membrane in a rat calvarial defect model [22,23]. Even though previous studies were not designed to study the osteoconductive properties of VSCM, they provide first insights towards this capacity [15,16]. The present study aimed at explicitly investigating the osteoconductivity of the porcine, porous, and cross-linked VSCM originally proposed for soft-tissue in a rat calvarial defect model.

## 2. Material and Methods

### 2.1. Study Design

Nine 7-month-old female Sprague Dawley rats from the Division for Biomedical Research (Himberg, Austria) were used. The animals were treated according to the guidelines for animal care; they were kept in groups of three animals each in Macrolon cages type IV with a day/night rhythm 1:1. Water and regular diet (ssniff Spezialdiäten GmbH, Soest, Germany) was provided *ad libitum*. The Medical University of Vienna ethical review board for animal research approved the study protocol (GZ BMWFW-66.009/0217-WF/V/3b/114/2012.20). The study was performed in 2019 at the Department of Oral Biology of the Medical University of Vienna in accordance with the ARRIVE guidelines [24]. The VSCM data are part of a larger study to evaluate the osteoconductive properties of collagen-based biomaterials but with a different hypothesis. It is thus legitimate to consider our data observed with a collagen membrane (Bio-Gide, Geistlich, Wolhusen, Switzerland) as a positive control considering the osteogenic properties of the rat calvaria defect [25,26].

### 2.2. Surgical Procedure

Two experienced periodontists performed the surgeries, and the operations were performed under general anesthesia to minimize the stress with ketamine 100 mg/kg (Pharmacia & Upjohn, Erlangen, Germany) and xylazine hydrochloride 5 mg/kg (Bayer, Leverkusen, Germany) by intramuscular injection. An incision was made in the center of the calvaria, and the periosteum was elevated to expose the cranial bony surface. One standardized circular defect was surgically induced on the rat calvaria unilaterally apart from the midsagittal suture, using a trephine drill with an external diameter of 5 mm (Dentium, Suwon, Korea). Prefabricated VSCMs of porcine origin (20 × 40 × 6 mm; Fibro-Gide, Geistlich, Wolhusen, Switzerland) were trimmed to cover the defects with sheets of size 10 × 10 × 3 mm. According to the manufacturer, the VSCM underwent chemical cross-linking. Wounds were closed with two layers of sutures (periosteum and skin) using resorbable silk (Vicryl 5-0; Ethicon, Norderstedt, Germany). For postoperative pain relief, butorphanol, 1.25 mg/kg (Richter Pharma AG, Wels, Austria) and meloxicam, 0.15 mg/kg (Boehringer Ingelheim Vetmedica GmbH, Ingelheim, Germany) were administered subcutaneously. The animals were postoperatively observed by the veterinarians of the Department of Biomedical Research of the Medical University of Vienna and by the surgeons who recorded possible behavioral problems, obvious pain, swelling, redness, or restricted mobility. One animal died during the healing period, the remaining nine animals were euthanized four weeks after surgery by an intracardial overdose of thiopental (Sandoz GmbH, Vienna, Austria).

### 2.3. Histological and Histomorphometric Analysis

The samples were dehydrated in an ascending alcohol series. Subsequently, they were embedded in light-curing resin (Technovit 7200 VLC + BPO; Kulzer & Co., Wehrheim, Germany). Blocks were further processed using EXAKT cutting and grinding equipment (Exakt Apparatebau, Norderstedt, Germany). Thin-ground sections from all samples were prepared in a plane parallel to the sagittal suture and through the center of the defect and stained with Levai–Laczko dye, composed of azure II and methylene blue, counterstain with safranin. The slides were scanned using an Olympus BX61VS digital virtual microscopy system (DotSlide 2.4; Olympus, Japan, Tokyo) with a 20× objective resulting in a resolution of 0.32 μm per pixel and then evaluated. For the histomorphometric analysis, the ROI contained the central defect area between the edges of the parietal bone and the ectocranial sides and above the defect area. The newly formed bone, the bone incorporating VSCM, the mineralized extracellular matrix resembling hypertrophic cartilage, the residual VSCM, and the soft tissue were measured. The different tissues were segmented manually on the computer screen at a high resolution and then exported into Fiji to obtain the correspondent histograms based on color-coded thresholding. 

### 2.4. Quantitative Backscattered Electron Imaging (qBEI)

One specimen that showed a particular strong bone formation was used for qBEI analysis. The details of the method can be found elsewhere [27,28] and are only shortly recapitulated here. To achieve a conducting surface, the sample was carbon-coated (Agar automatic SEM carbon coater, Agar Scientific Ltd., Stansted, Essex, UK) and subsequently scanned with a field emission scanning electron microscope SUPRA40 (Zeiss, Oberkochen, Germany). Prior to measurement, the device was calibrated with aluminum and carbon standards allowing for the quantitative measurement of the local calcium content. Bit grey level images with pixel resolution 0.882 µm were acquired. The obtained grey values were then converted in calcium concentration in weight percent (wt.% Ca) and the bone mineralization density distribution (BMDD) was evaluated. The BMDD is the frequency distribution of local calcium content normalized to 100 % bone area [27]. The BMDD curve is characterized by the following parameters: Ca_Peak_, the most frequently measured calcium concentration; Ca_Mean_, the mean calcium concentration; and Ca_Width_, the heterogeneity of the mineralization given by the full width at half maximum of the curve. 

### 2.5. Micro-CT Analysis

Whole crania of animals were retrieved and the samples were fixed in phosphate-buffered formalin (Roti-Histofix 4%; Carl Roth, Karlsruhe, Germany). Micro-CT scans were performed with a SCANCO μCT 50 (SCANCO Medical AG, Bruttisellen, Switzerland) at 90 kV/200 μA with an isotropic resolution of 20.1 μm and an integration time of 500 ms. Three-dimensional isosurfaces of the collected data were processed using Amira 6.2 (Thermo Fisher Scientific, Waltham, MA, USA). The images were rotated using Fiji [29] such that the drill direction was oriented along the Z-axis with the defect in the approximate center of the image. The regions of interest (ROIs) were defined manually, comprising the areas within and above defect area (defect and above), and segmented automatically by setting the threshold of 450 mgHA/cm^3^ to distinguish the mineralized tissue from the background. The newly formed bone connected to the circumferential bone and the bone coverage were measured. Calibration and blinding of the examiner were not necessary as the software performed the analysis automatically using the specifically developed Fiji rule set which was identical for all samples. The rule set is made available on request. 

## 3. Results

### 3.1. Histologic Results

**Figure 1:**
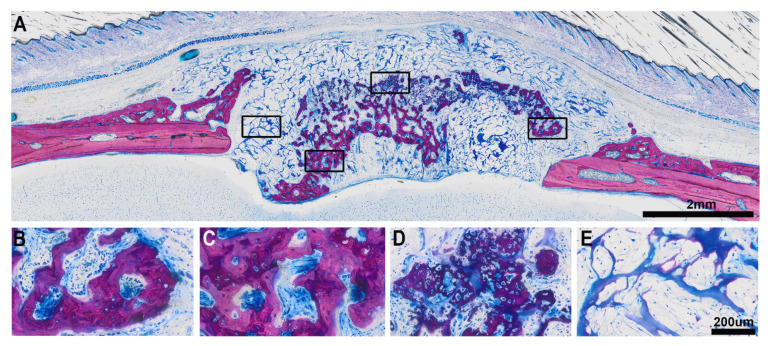
Sagittal section of the augmented calvaria going through the defect. (**A**) The overview shows the formation of mineralized tissue inside the VSCM that bridges the defect. The mineralized tissue is heterogeneous in appearance. (**B**) The picture shows the characteristic osteoblasts seams with the unmineralized osteoid. Part of the new bone entombs the VSCM fibers that appear pink. (**C**) The new woven bone also appears without any visible VSCM fibers. (**D**) We further observed a mineralized extracellular matrix that resembles a hypertrophic cartilage-like tissue and (**E**) areas of VSCM fibers appearing as blue connected islands with a void space filed by loosely distributed cells. Scale bars: 2 mm (**A**) and 200 μm (**B**–**E** have the same magnification).

**Figure 2:**
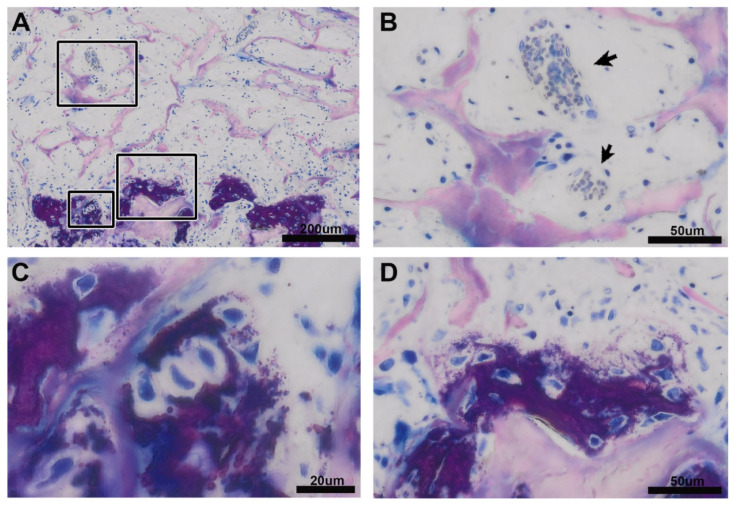
Mineralized extracellular matrix that looks like a hypertrophic cartilage-like tissue. What becomes obvious is that the scattered mineralized tissue appears at a certain distance to the blood vessels (**A**). The blood vessels (black arrows) have sprouted into the VSCM stained in light pink and are characterized by the erythrocytes that appear in light blue stain (**B**). Impressively is the morphology of the mineralizing cells that occasionally form staples, the hallmarks of hypertrophic chondrocytes in the growth plate (**C**) but are also loosely distributed in their mineralized matrix (**D**). The strong uptake of the dye produces a dark purple stain that indicated higher mineralization of the extracellular matrix compared to the pink staining of the classical newly formed bone that is produced by the osteoblasts.

**Figure 3:**
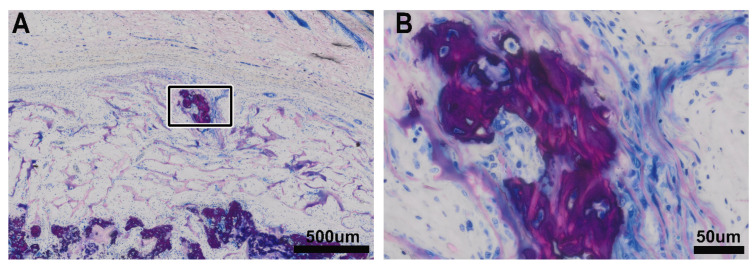
Mineralized VSCM. We also observed areas where the pink collagen bundles of VSCM were embedded in the dark purple newly formed mineralized matrix (**A**), again showing the morphological features of hypertrophic chondrocytes with the large lacunae and independent of an osteoid seam that indicates the functional activity of osteoblasts. (**B**) These mineralized islands can be next to the outer surface of the VSCM indicated by the paralleled and elongated fibroblastic cells that are independent and separated from the main front of mineralized tissue formation.

**Figure 4:**
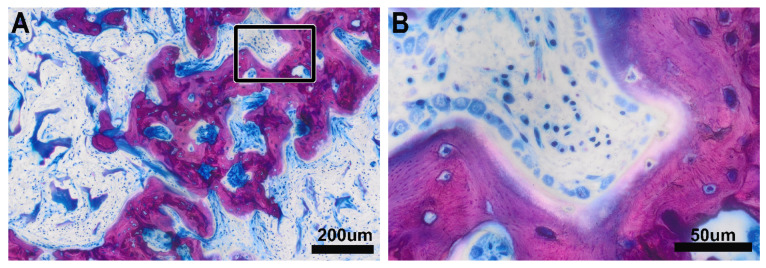
New bone in the defect area. Typical morphology of the newly formed bone with the different shades of dark pink staining of a bone that forms within the VSCM (**A**). The ultimate hallmarks of bone are the osteoblastic seams separated by the osteoid layer from the mineralization zone (**B**). The osteocytes are embedded in the newly formed osteoid layer. The morphological appearance of the newly formed bone is in sharp contrast to the areas considered as mineralized chondrogenic cells.

**Figure 5:**
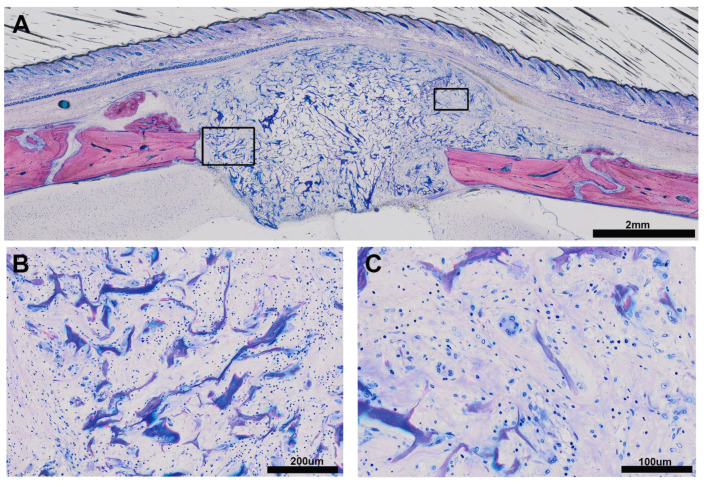
Insights into the VSCM show no mineralization. The specimen that shows no bone formation is depicted in the overview (**A**). The presence of inflammatory infiltrate surrounding the VSCM fibers is visible (**B**). A higher magnification view reveals occasionally multinucleated cells and light purple staining that indicated the presence of newly formed extracellular matrix produced by spindle-shaped fibroblastic cells (**C**).

The histological analysis identified a heterogeneous pattern of bone formation within the implanted VSCM (Figure 1, Figure 2, Figure 3 and Figure 4) in eight specimens (A); one specimen had no bone formation penetrating into the VSCM (Figure 5). Highly magnified views demonstrated two types of mineralized tissue formation: (i) the expected new woven bone with the characteristic osteoblast seams and (ii) clusters of a highly scattered mineralized extracellular matrix resembling hypertrophic chondrocytes during development [30,31] and endochondral fracture healing [32]. The differentially stained woven bone appears (B) with pink VSCM fibers entombed or (C) without VSCM. (D) Unexpected large mineralized areas resembling hypertrophic cartilage can be distinguished from the woven bone by means of morphology. (E) Areas exclusively occupied by VSCM fibers that appear bluish are also visible.

### 3.2. Histomorphometric Results

**Figure 6:**
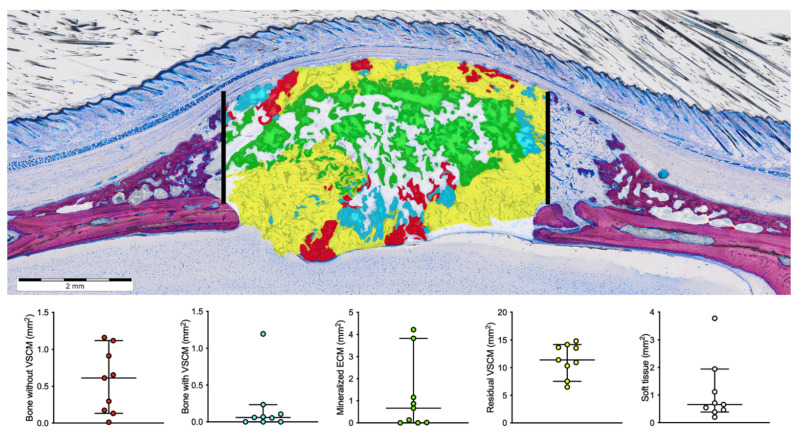
Histomorphometric analysis—strategy of segmentation and descriptive statistics. The segmentation was based on the characteristic features of tissues by high-resolution microscopy. Manual segmentation depicts the new bone alone without VSCM (red), the new bone entombing the VSCM (cyan), the extracellular matrix resembling mineralized cartilage (mineralized ECM; green), the VSCM alone (yellow), and the remaining soft tissue (white). The bars show the median and 95% confidence interval.

This analysis was based on the manual segmentation of tissue with a characteristic morphology as indicated by Figure 6 showing the false-color staining in the various ROIs. The histomorphometric analysis identified newly formed bone without embedded VSCM to be in median 0.67 mm^2^ (0.011 min; 2.4 max) while the new bone with embedded VSCM was only 0.06 mm^2^ (0.0 min; 1.2 max). The mineralized extracellular matrix resembling hypertrophic cartilage was 0.66 mm^2^ (0.0 min; 4.2 max). Most of the area was occupied by the remaining VSCM with no mineralized tissue (11.4 mm^2^, 6.5 min; 14.8 max). 

### 3.3. Quantitative Backscattered Electron Imaging (qBEI)

**Figure 7:**
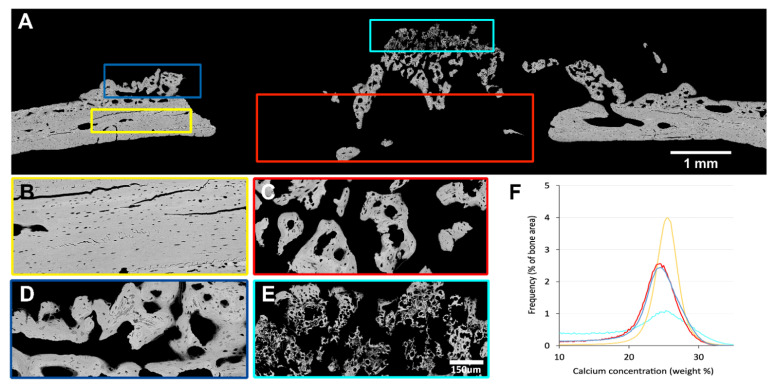
Quantitative backscattered electron imaging (qBEI) of one representative specimen. (**A**) The overview shows the regions of interest, which are prepicked, (**B**) in yellow for pre-existing cortical bone, (**C**) in red for new bone within the defect, and (**D**) in blue next to the periosteum, (**E**) in cyan the mineralized extracellular matrix with its scattered appearance of hypertrophic chondrocytes. The high magnification pictures correspond to these ROI showing the homogenous distribution of mineral in the pre-existing cortical bone, the various shades of grey in the immature new bone in the defect sites, originating from the periosteum and new bone observed inside the defect. Of particular interest is the heterogenous appearance of the mineralized extracellular matrix that closely resembles hypertrophic cartilage during development or upon fracture healing. (**F**) The bone mineral density-distribution obtained for the four different depicted ROIs; cortical bone (yellow) is higher mineralized than new bone (red and blue), and new bone in both investigated locations (within the defect and close to the periosteum) has a similar mineral distribution. In contrast, the scattered mineralized matrix with hypertrophic chondrocytes shows a very heterogenous mineral distribution (cyan). Scale bars: 1 mm (**A**) and 150 μm (**B**–**E** have the same magnification).

For further characterization, backscattered electron microscopy was performed. As expected, the newly formed woven bone (blue and red ROI) is lower and more heterogeneously mineralized than the pre-existing cortical bone of defect margins (yellow ROI) (Figure 7 Table 1). This is shown by the low Ca_Peak_ and Ca_Mean_ and the large Ca_Width_ for woven bone compared to the pre-existing cortical bone of the calvaria. Moreover, there is no significant difference in mineralization between newly formed bone connected to the pre-existing cortical bone (blue ROI) and inside the defect (red ROI). In contrast, the chondrogenic mineralized extracellular matrix has a remarkably distinct mineralization pattern and different morphology compared to the other tissue types (cyan ROI). The mineralization is heterogeneously combining a large portion of lowly mineralized bone, but also regions that are higher mineralized than the other tissues (Table 1).

The cortical bone shows high and homogenous mineralization, the new bone is less mineralized than the cortical bone. The mineralized extracellular matrix shows a rather high mineralization peak but a wide distribution range and a lower mean calcium content than cortical and new woven bone.

### 3.4. Microtomographic Findings

**Figure 8:**
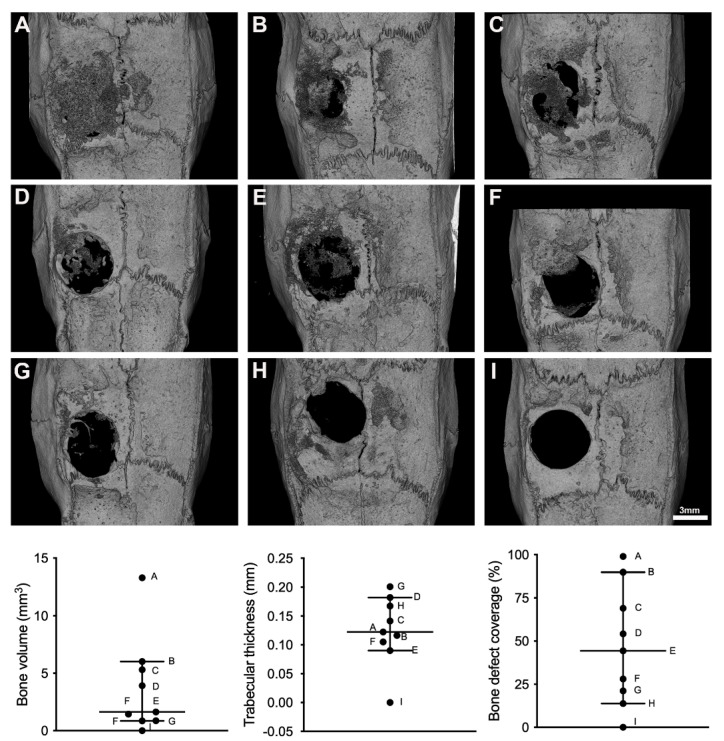
MicroCT analysis of defect coverage. The images show the defect viewed from above. The partial mineralization of the defect is visible. Within the regions of interest representing the defect margins, we measured the bone volume in mm^3^, the mean trabecular thickness in mm, and the defect coverage in %. Please note the large variation of the newly formed mineralized tissue within the nine specimens. The bars show the median and 95% confidence interval.

In line with the histomorphometric data, hyperdense mineralized tissue was observed in eight out of nine samples (Figure 8). The overall bone volume in the defect was in median 1.6 mm^3^ (min 0.0; max 13.3), the mean trabecular thickness was 0.12 mm in median (min 0.0; max 0.2). One defect was almost fully covered with new bone tissues but there was a wide range in the coverage of the defect with a median of 44.4% (min 0; max 99%). 

## 4. Discussion

VSCMs serve as scaffolds for soft tissue augmentation around teeth or dental implants [10,11,12,33]. Apart from serving as a space-maintaining scaffold to augment the soft tissue, VSCM allows for bone formation originating from the underlying ridge defect [15] and supports periodontal regeneration in a canine model [16]. However, studies to explicitly evaluate the osteoconductive properties of a VSCM in a preclinical setting are still missing. Therefore, the present pilot study combines different analytical methods to determine the osteogenic consolidation of a VSCM in rat calvarial defects. We found different types of mineralized tissues within the VSCM consisting of woven bone and a heterogeneously mineralized extracellular matrix resembling hypertrophic cartilage. VSCM fibers entombed within the new bone were only occasionally observed. The osteogenic consolidation of a VSCM, however, was not predictable as indicated by the large variety of bone formation within each specimen and between the individual specimens. Nevertheless, the present study confirms existing evidence for the possibility of new bone formation within the VSCM, thus confirming and extending the first reports showing little bone formation inside the VSCM in a dog model [15,16]. VSCM was also reported to be encapsulated by soft tissue without any evidence of mineralization [15,34]. Consistent with the previous observations, VSCM is not an ideal material to support bone formation and might be developed towards a biomaterial supporting chondrogenesis.

Further support for this suggestion comes from our observations that VSCM favors the formation of a mineralized extracellular matrix, that resembles the hypertrophic cartilage-like tissue of growth plates and ossification centers during development [31] and the fracture callus [32]. We found aggregated small mineralization nodules surrounding the cells that featured completely separated but randomly dispersed spheroidal-shaped cells with a halo. This mineralized extracellular matrix was distinguished from the typical woven bone formation i.e., apart from the morphology, by the differential staining intensity and the mineralization distribution. This endochondral healing pattern might be caused by the sponge-like characteristic of VSCM that is mechanically unstable and slowly vascularized, both conditions favoring the differentiation of mesenchymal cells into chondrocytes [35]. 

Even though VSCM failed to release detectable amounts of TGF-β activity into an aqueous extraction [36], the endogenous TGF-β may support the chondrogenic differentiation of the invading mesenchymal cells [36,37]. The role of TGF-β to support chondrogenic differentiation remains at the level of a hypothesis. Nevertheless, it is the collagen membranes that are used in cartilage repair serving as a matrix for the mesenchymal cells originating from the bone marrow termed “autologous matrix-induced chondrogenesis” [38] or directly seeded onto the membranes [39]. Thus, it might be possible that VSCM can be modified in a way to be used for cartilage repair where the transplanted cells usually undergo fibroblastic differentiation [40,41]. 

If we relate the findings to those of others, cartilage-like tissue formed in the pores of copolymer scaffolds not containing TGF-β when transplanted into rat calvaria defects [42]. This is maybe not surprising as calvaria-derived cells are rich in mesenchymal progenitors with the capacity to differentiate into the chondrogenic lineage under the appropriate conditions [43]. There is also solid experimental evidence that by increasing the stiffness of gelatin scaffolds chondrogenesis and thus endochondral ossification is promoted [17,18]. It seems that some scaffolds and matrices favor this differentiation process, an observation that leads to new research questions. Our findings and those of others may also inspire the emerging field of bioprinting allowing us to adapt the properties of collagen to be applied in various clinical indications including bone but also cartilage regeneration [44].

We acknowledge several limitations of our study. There is strong but indirect evidence for the presence of endochondral bone formation, similar to the formation of cartilage upon sinus augmentation [45]. It is the characteristic morphology, in combination with the typical strong staining of cartilage by the Levai-Laczko [46], together with the shift in the mineral distribution that points towards the formation of a hypertrophic mineralized cartilage inside the VSCM. Moreover, considering that also endochondral bone formation during development and fracture healing is a continuous process, we observed a gradient where the mineralized hypertrophic chondrocytes are adjacent to the new bone, further supporting the impression of an endochondral bone formation. To further strengthen this hypothesis expression of cartilage markers such as collagen type X and matrix metalloproteinase 13 could be measured [47]. 

One might also consider a limitation that we have only included one specimen for the qBEI analysis; that specimen, however, showed the characteristic heterogeneous appearance of the mineralized extracellular matrix that closely resembles hypertrophic cartilage. The qBEI was not a primary endpoint but only to better understand the mineralization pattern of this what we think is endochondral bone formation. Considering this limitation though, the mineral distribution of the pristine and the new woven bone is different from the endochondral bone formation. Another limitation is that no negative controls have been performed. Yet, we have positive controls as this research is part of a larger series of experiments testing the osteoconductive properties of collagen membranes [25,26]. We have observed an almost complete defect coverage when using a native collagen membrane (Bio-Gide, Geistlich, Wolhusen, Switzerland) as a positive control in rat calvaria defects [25,26]. This control is valid for the surgical technique and also the osteogenic capacity of the calvaria defects. It is thus legitimate to conclude that the VSCM behaves differently when compared to the native collagen membrane used for guided bone augmentation. Considering that the studies have a different research question, the data were reported elsewhere [25,26]. Another appropriate control would be a native non-cross-linked VSCM to understand how the stiffness of the biomaterial affects its chondrogenic properties.

From a clinical perspective, it would be desirable to modify the VSCM to preserve the volume stable properties but having also the osteoconductive properties of a porcine collagen membrane [20,21]. Thus, the results of the present study provide new and unexpected insights into the osteogenic properties of a VSCM and also raise new research questions that inspire the further development of osteoconductive scaffolds and their clinical application. At least in the rat calvaria defect model, VSCM shows a highly variably outcome in terms of mineralization also indicating a limited capacity to support intramembranous bone formation but at least allows the formation of bone via the endochondral route. This observation should not be clinically overinterpreted but may serve as a primer to increase our understanding of how material properties affect the fate of mesenchymal cells towards the osteogenic or the chondrogenic lineage. 

## Figures and Tables

**Table 1:** Quantitative backscattered electron microscopy.

	Ca_Mean_ (wt % Ca)	Ca_Peak_ (wt % Ca)	Ca_Width_ (Δ wt % Ca)
Cortical bone	24.9	25.5	3.8
New bone periost	22.2	24.4	5.6
New bone defect	22.1	24.4	5.2
Mineralized ECM	17.8	25.1	9.2

## Data Availability

Not applicable.
